# Precision medicine for hepatocelluar carcinoma using molecular pattern diagnostics: results from a preclinical pilot study

**DOI:** 10.1038/cddis.2017.229

**Published:** 2017-06-08

**Authors:** Rahul Agarwal, Yuan Cao, Klaus Hoffmeier, Nicolas Krezdorn, Lukas Jost, Alejandro Rodriguez Meisel, Ruth Jüngling, Francesco Dituri, Serena Mancarella, Björn Rotter, Peter Winter, Gianluigi Giannelli

**Affiliations:** 1 GenXPro GmbH, Frankfurt am Main, Germany; 2 IRCCS 'S De Bellis', National Institute of Gastroenterology Castellana Grotte, Italy

## Abstract

The aim of this study was to design a road map for personalizing cancer therapy in hepatocellular carcinoma (HCC) by using molecular pattern diagnostics. As an exploratory study, we investigated molecular patterns of tissues of two tumors from individual HCC patients, which in previous experiments had shown contrasting reactions to the phase 2 transforming growth factor beta receptor 1 inhibitor galunisertib. Cancer-driving molecular patterns encompass – *inter alias* – altered transcription profiles and somatic mutations in coding regions differentiating tumors from their respective peritumoral tissues and from each other. Massive analysis of cDNA ends and all-exome sequencing demonstrate a highly divergent transcriptional and mutational landscape, respectively, for the two tumors, that offers potential explanations for the tumors contrasting responses to galunisertib. Molecular pattern diagnostics (MPDs) suggest alternative, individual-tumor-specific therapies, which in both cases deviate from the standard sorafenib treatment and from each other. Suggested personalized therapies use kinase inhibitors and immune-focused drugs as well as low-toxicity natural compounds identified using an advanced bioinformatics routine included in the MPD protocol. The MPD pipeline we describe here for the prediction of suitable drugs for treatment of two contrasting HCCs may serve as a blueprint for the design of therapies for various types of cancer.

## 

Hepatocellular carcinoma (HCC) is one of the most lethal cancers worldwide. Nearly 745 000 people died from it only in 2012.^1^ Patients' 5-year overall survival (OS) rate of <20% indicates the urgent need for alternative therapies to improve the outcome for these patients.^2^ HCC develops along different clinical histories including chronic hepatitis, cirrhosis and alcoholism.^3^ All these factors contribute to unceasing inflammation and regeneration of hepatocytes, making it challenging to achieve diagnosis and prognosis of HCC at earlier stages.

Currently, the multikinase inhibitor sorafenib is the only effective, approved systemic therapy for advanced HCC that are not suitable for other curative treatment,^4^ but the occurrence of side effects has markedly reduced the impact of the drug in daily life clinical practice.^5, 6^ Given the limited efficiency of the standard treatment, as well as the occurrence of drug resistance,^7^ we addressed the question whether the newly arising concept of precision oncology could enable us to design novel therapeutic strategies that take into account the genetic diversity of these patients' tumors.

Other drugs such as the transforming growth factor beta receptor 1 (TGFBR1) blocker galunisertib are undergoing clinical trials for the treatment of HCC.^8^ A large body of evidence indicates that TGFB1 is an important key to tumor progression, as it promotes the epithelial-to-mesenchymal transition (EMT) and activates the WNT pathway, a hallmark of HCC.^9^

Immune therapy is currently being considered for the treatment of HCC and a comprehensive meta-analysis of recent studies encompassing more than 1800 patients indicates that patients undergoing specific immunotherapy benefit from a significantly higher overall and recurrence-free survival than those in control groups.^10^ TGFB1 plays an important role in the regulation of immune responses via cancer-associated fibroblasts (CAFs) that express the growth factor in a self-sustaining autocrine cycle. CAFs sustain oncogenic features of cancer cells including suppression of the functions of various immune cells, particularly effector T cells and natural killer (NK) cells. TGFB1 also regulates T-regulatory cells (Treg) maturation and thereby suppresses immune responses.^11^ As in other cancers there is ample evidence that also in HCC, control of the immune system by the neoplastic complex contributes significantly to the survival of cancer cells. It has been shown, for example, that the presence of a certain dysfunctional subset of tumor-infiltrating NK cells is associated with tumor progression and is an independent indicator of poor outcome in HCC patients.^12^

Recent work indicates that patients suffering from refractory cancers that were treated by genomics-guided precision medicine did indeed have a significantly better progress-free survival (PFS) ratios and longer median PFS compared with patients who did not receive personalized therapy.^13^ Precision oncology has been facilitated by the advent of next-generation sequencing, which enables particular molecular genetic profiles to be identified in the individual patient who may be targeted by precise, personalized therapy. Identified targets are then used to search databases for drugs that address these aberrantly expressed molecules and pathways using the bioinformatics pipeline. This concept benefits from the fact that drugs have been developed and are applied for many molecular targets, across a plethora of different diseases. The knowledge of the individual architecture of a patient’s cancer may now enable these drugs to be directed against these specific oncogenic features, in a form of one-person trial. Drug repurposing, retasking or reprofiling has already been demonstrated as a promising strategy for cancer therapy,^14^ which could be justified if the patient’s tumor reveals molecular patterns indicative of an altered mitochondrial function, like the Warburg effect. Thus, given that suitable targets can be identified for the individual cancer, readdressable drugs are often at hand to attack it.

Recently, we have characterized HCC tissues by their differential response to galunisertib^15^ using NGS-based massive analysis of cDNA ends (MACE),^16, 17^ high-throughput transcription profiling for the investigation of aberrant signaling and metabolic pathways and all-exome-sequencing for the identification of somatic mutations accompanying neoplasia.^18^ Based on this comprehensive set of data, molecular pattern diagnostics (MPDs) first identify biological pathways most enriched in overexpressed genes in the tumor. MPDs then help to select the potentially best suited drugs according to the number of targeted enriched pathways, resulting in a ranked list of drugs suited for approved use or repurposing in the frame of a monotherapy or for combination therapies with other immune-related drugs or natural compounds.

The aim of this study was to design a road map for precision medicine of HCC by using MPD for the analysis of two HCC tumor tissues that responded differently to galunisertib treatment.

## Results

To investigate the molecular targets and druggable pathways for patients with HCC, we performed MACE genome-wide transcriptome sequencing and Exome-Seq all exome sequencing of tumoral (called 'tumor') and peritumoral (called 'normal') tissues extracted from tumors that had previously been shown to react differentially to TGFB1 and to the TGFBR1 inhibitor galunisertib.^15^

**Figure 1:**
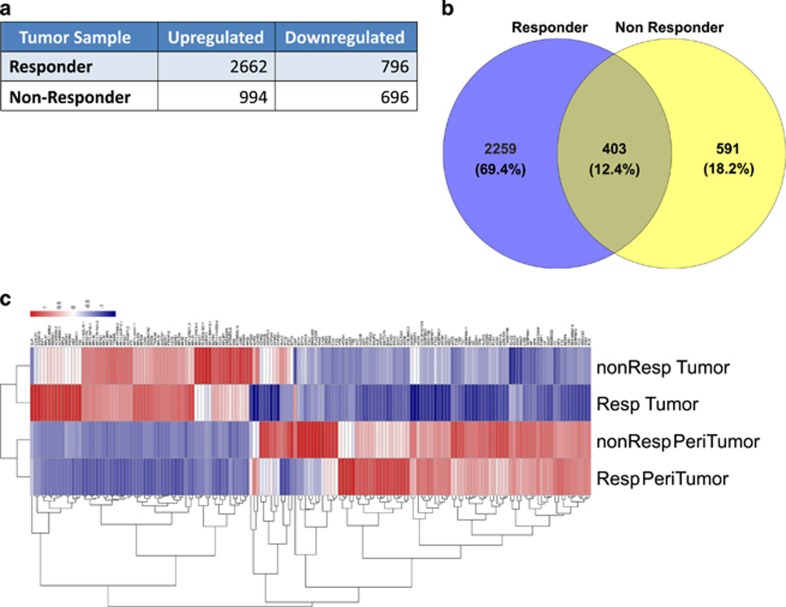
Up- or downregulated genes in HCC tissues after galunisertib treatment. In (**a**), the total number of significantly (log 2FC >2 and *P*-value <0.01) up- or downregulated genes in responder- and non- responder tumor tissues, in comparison with corresponding normal tissues. In (**b**), the total number of overexpressed genes in both responder and non-responder tumor tissues compared with their corresponding normal tissues. The Venn diagram presents the total number of dissimilar and shared genes in responder and non-responder tumor tissues based on the total number of differential genes. Within parentheses, their percentages are given in relation to the aggregate number of differentially expressed genes in both tumor tissues. In (**c**), MACE profiles of responder- and non-responder normal and tumor tissues differentiating between all tissues. Unsupervised hierarchical cluster analysis of 164 strongly expressed genes (based on high intensities (50% >100) and high variability (interquartile range (IQR)>1.5)) clearly separates normal and tumor tissues. Normalized expression values are rescaled as shown in the sidebar, where a positive number (red) indicates high expression and a negative number (blue) low expression

Using MACE we found a large number of genes differentially expressed between normal and tumor samples of both patients. In comparison with the non-responder tissue, there were relatively higher number of significantly (absolute log 2 fold change (log 2fc) >2 or <−2 and *P*-value <0.01) differentially expressed genes in the responder sample, as depicted in Figure 1a. A total of 403 genes were similarly upregulated in both tumors in comparison with both normal tissues (Figure 1b). Unsupervised hierarchical clustering of strongly expressed genes shows a clear distinction between the transcriptomes of normal and tumor tissues (Figure 1c). Principal component 1 explained 79.8% of the total variance in the entire transcriptome data estimated by PCA analysis of 164 strongly expressed genes (data not shown).

**Figure 2:**
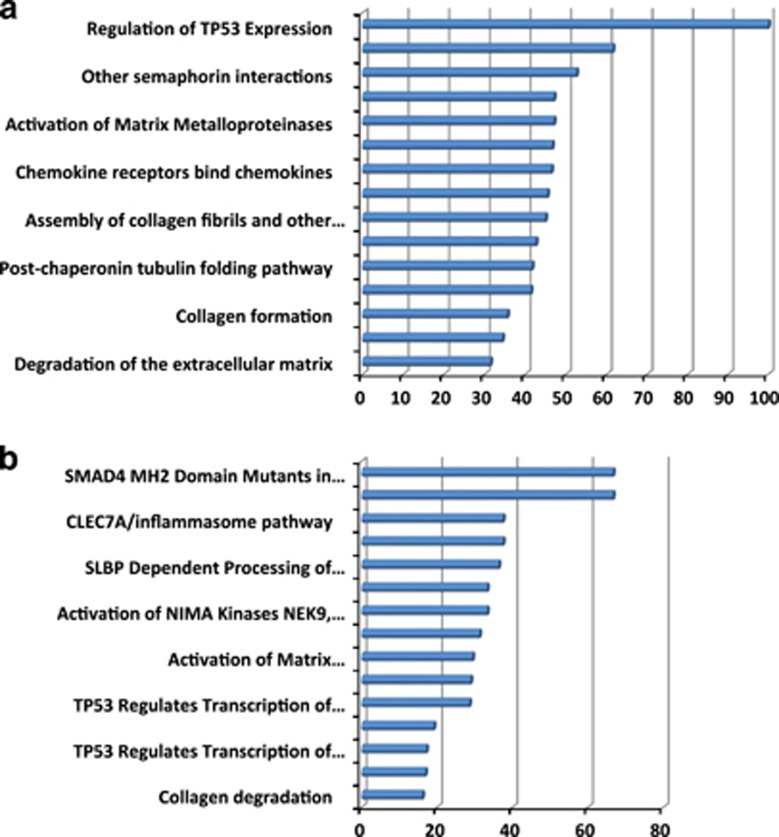
Percentage of upregulated genes in responder tissues after galunisertib treatment. Percentage of upregulated genes in comparison with all genes in the pathway, in the top 15 reactome pathways most enriched in upregulated genes in the responder tumor tissues in comparison with its normal reference tissue (**a**). Percentage of upregulated genes in comparison with all genes in the pathway in the top 15 reactome pathways most enriched in upregulated genes in the non-responder tumor tissue in comparison with its normal reference (**b**)

Genes differentially expressed in both responder and non-responder tumor samples in relation to their respective normal samples were subjected to Gene Ontology and Reactome pathway analyses to identify molecular processes and pathways differentiating normal from neoplastic tissues. The most significant pathways that clearly differentiate the two tumors from their respective reference tissues are shown in Figure 2.

In the responder tumor all four genes in the reactome pathway 'regulation of TP53 expression' including PRDM1 (known also as BLIMP1) were upregulated. Thus, upregulation of this pathway is involved in abnormal growth of these cancer cells. The transcriptome of the responder tumor also showed enriched pathways related to the formation, degradation and remodeling of the extracellular matrix (ECM) indicated by reactome pathways 'dissolution of fibrin clot', 'degradation of the extracellular matrix', 'activation of matrix metalloproteinases', 'collagen degradation' and 'non-integrin membrane–ECM interactions'. These pathways together included the largest number of upregulated genes and may be crucial for cell migration and evasion of cancer cells from the tumor and hence metastasis. In the non-responding tumor, enrichment of the pathways 'loss of function of SMAD4 in cancer' and 'SMAD4 MH2 domain mutants in cancer' indicates that the non-responder tumor shows impaired TGFB1-related transcription initiation, which corresponds to the observed (non-responder) phenotype. More than 70% of the genes in these pathways are upregulated in the tumor. Further, the transcriptome of the non-responding tumor tissue showed – like that of the responder tumor – some enriched ECM-related pathways (e.g. 'collagen degradation', 'activation of matrix metalloproteinases'). Other pathways such as 'PERK regulates gene expression' and 'ATF4 activates genes' indicate marked membrane stress and unfolded protein responses. Cell-cycle control by P53 and EMI1 is indicated by the enrichment of the pathways 'TP53 regulates transcription of cell cycle genes' 'TP53 regulates transcription of genes involved in G2 cell cycle arrest' and 'phosphorylation of EMI1'. Thus, upregulation of the NIMA kinase pathway may be the upstream signal for the observed induction of the immune response-related pathways 'CLEC7A/inflammasome pathway' and 'interleukin-19 (IL-19), -20, -22 and -24' that indicate enhanced innate defense and tissue repair processes in the non-responder tumor.

**Figure 3:**
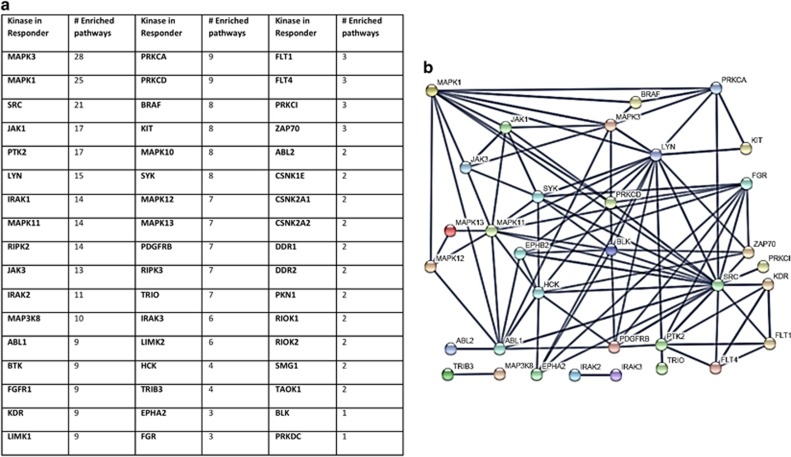
Kinases upregulated in responder tissues after galunisertib treatment. List of kinases engaged in upregulated reactome pathways in the responder tissue. The number of pathways in which the kinases are active is also indicated (**a**). STRING network of kinases engaged in upregulated reactome pathways in the responder tumor. Only interactions of the highest confidence level are shown. Kinases that do not interact with sufficient security are omitted (**b**)

As depicted in Figure 3a in the responder tissue, there are more than 50 upregulated kinases engaged in at least one upregulated reactome pathway. To investigate whether these kinases would interact with each other and to help with selecting, especially promising drug targets with large numbers of interactors, we performed a STRING analysis of protein–protein interactions of upregulated kinases in the responder tumor. This analysis delivered a kinase interaction network in which i. A. SRC, MAPK1, FGR, SYK and LYN form strongly interconnected hubs (Figure 3b).

Since kinase activity is usually regulated by phosphorylation and not by overexpression, upregulation of a kinase hints at an even more important role in cancer development and maintenance. The MPD pipeline offers the options to select drugs according to general reactome profiles and/or to focus on particular pathways such as immune-related pathways that are already suggested by their molecular signatures. The first option interrogates the Human Protein Atlas (http://www.proteinatlas.org) database containing ~670 FDA-approved drugs targeting functions enriched in upregulated genes and the respective proteins facilitating that function. The complete list resulting from relating the responders tumors' individual molecular patterns to this database is depicted in Table 1.

A list of kinases that are upregulated in the responder tissue are reported in Table 2. Several of them are targeted by approved or experimental tyrosine kinase inhibitors in different stages of development, as well as by natural, low-toxicity anticancer compounds.

Correlating drug reactivity profiles to enriched reactome pathways for the non-responder yielded the list of drugs depicted in Table 3. The first drug on the list is marimastat, which blocks several matrix metalloproteinases (MMPs) that are upregulated in the tumor tissue. Since MMPs are needed for the dissolution of the ECM enabling cancer cells to leave the tumor, their inhibition could help to prevent metastasis. The COX-2 inhibitor celecoxib, however, which was developed for treating rheumatic and degenerative diseases, also inhibits a set of at least six proteins that are upregulated in the tumor.

As depicted in Table 4, the number of kinases engaged in upregulated pathways in the non-responder tissue and resulting STRING protein interaction network (not shown) is much smaller than in the responder tumor. Consequently also the number of kinase inhibitors potentially available for treatment of the tumor is reduced. The small list of upregulated kinases in the non-responder tumor (Table 4) delivers only a few addressable targets. There are three approved drugs available for inhibition of MAP2K6 that is engaged in three enriched pathways and also MAPK11 that functions in two enriched pathways is targeted by Regorafenib. However, whereas the latter drug targets several kinases of expected relevance in the responder tumor, it only targets one (MAPK11) in the non-responder tumor tissue. Additional studies are needed to learn whether the drug would have similar effects in both patients.

The upregulation of different immune-related pathways in both tumors (Table 5) indicates a potential for tailored treatment alternatives or amendments to pathway inhibitors by addressing the immune checkpoints in the two tumors. Therefore, in Table 5, we present a list of immune-related pathways enriched in upregulated genes in the two tumors.

In the responder tumor the 'PD-1 signaling' pathway is most prominent, indicating that immune responses in the tumor are under the control of the immune checkpoint inhibitor PD1. By contrast, the non-responder tissue does not seem to rely on this type of control of the immune system since none of the PD1 signaling pathway genes are upregulated (Table 5). The second most upregulated immune pathway in the responder tumor is 'regulation of innate immune response to cytosolic DNA', which corresponds to the concomitantly upregulated 'stimulator of interferon gene (STING)-mediated induction of host immune responses' pathway also dealing with the modulation of immune responses by cytosolic DNA. STING is a cytosolic receptor that senses both exogenous and endogenous cytosolic DNA. It activates TANK-binding kinase 1/interferon regulatory factor 3, nuclear factor-*κ*B (NF-*κ*B) and signal transducer and activator of transcription 6 (STAT6) signaling pathways to induce robust type I interferon and proinflammatory cytokine responses. The STING signaling pathway can be targeted by STINGVAX. STINGVAX are cyclic dinucleotides that are ligands formulated with granulocyte–macrophage colony-stimulating factor-producing cellular cancer vaccines. 'cytotoxic T-lymphocyte-associated protein 4 (CTLA4) inhibitory signaling' is another pathway that is highly enriched in upregulated genes in the responder tissue. The CTLA-4 is the inhibitory checkpoint receptor on T cells. CTLA-4 upregulation by certain Tregs is linked to host immune tolerance after liver transplantation.

In the non-responder tumor the 'CLEC7A/inflammasome pathway' is the most prominent dysregulated pathway potentially available for therapeutic intervention (Table 5). CLEC7A (also called dectin-1) is located on the surface of particular immune cells. It belongs to a class of C-type lectin pattern recognition receptors that are involved in immune responses to different pathogens.

Besides MACE transcription profiling we performed comparative all-exome sequencing of the corresponding tumor and normal tissues of the responder and non-responder tumors to disentangle mutations that may eventually help to explain the differential gene expression in the two tumors. Moreover, information about mutated genes might also serve to avoid administering drugs targeting non-functional or irreversibly activated kinases and phosphatases. The number of SNPs/InDels in the tumor tissues *versus* the respective peritumoral tissue according to their genomic location in relation to genes is summarized in Table 6.

**Figure 4:**
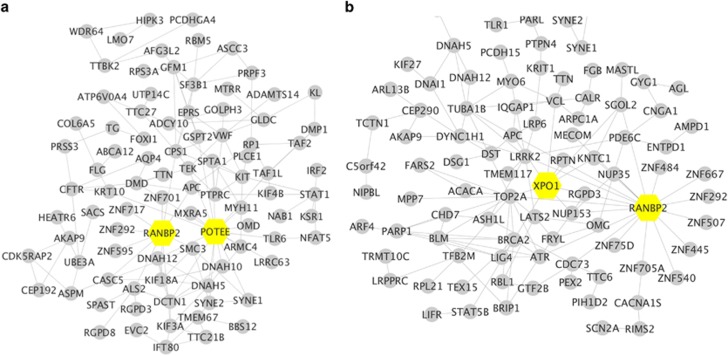
STRING interaction network in HCC tissues treated with gaunisertib. STRING interaction network of proteins altered by non-synonymous mutations in the responder tumor tissue, panel (**a**), and in the non-responder tumor tissue, panel (**b**)

To identify potential mutated hub genes within the tumors that might interact with other mutated genes we again performed a STRING protein–protein interaction network analysis of genes carrying protein-changing mutations, Figure 4a (responder) and B (non-responder).

Like the responder interactions, also the non-responder STRING protein interaction network shows the RANBP2 protein as a central hub in the network of mutated proteins. As in the responder, also in the non-responder it interacts with several mutated zinc-finger proteins (ZNF 507, 484, 75D, 667, 540, 292, 750A) (Figure 4), although these differ from those of the responder. In addition, the *TOP2A* gene, another hub gene in the network with which it interacts, is also mutated in the non-responder. RANBP2 sumoylates the TOP2A protein in mitosis, which is required for the proper localization of TOP2A to centromeres.

Our assumption that TGFB function is impaired in the non-responder tumor is not only based on its phenotype but also on the fact that this tumor carries a nonsynonymous C to A mutation in the *TGFBR3* gene (also known as betaglycan) on chromosome 1 at position 91 716 677 (*P*-value=0.000980), which, to our knowledge, has not been previously described in HCCs. Contrary to TGFBR1 and TGFBR2, which bind directly to some TGFB superfamily ligands, TGFBR3 is a coreceptor not only for TGFB but also for related factors such as activins, inhibins, growth differentiation factors and bone morphogenetic proteins. To answer the question whether in our particular (non-responder) tumor TGFB3 may also interfere with EMT, we took a closer look into the expression of TGFB-related transcripts in both tumors. In the responder tumor and non-responder tumor in which we list TGFB pathway transcripts that are significantly differentially expressed (log 2fc⩾2), we found much more upregulated transcripts in the responder (61) than in the non-responder tumor (23). Especially in the responder tumor overexpressed transcripts include the growth factors TGFB1 (log 2fc=−2.07) and TGFB1/1 (log 2fc=−3.27) themselves, as well as their direct pathway targets SMAD3 (log 2fc=−2.44) and SMAD7 (log 2fc=−3.86). Other upregulated genes in the pathway include TP53 (log 2fc=−2.43), SRC (log 2fc=−4.82) and STAT1 (log 2fc=−2.35). The ZEB1 (log 2fc=−2.28) and ZEB2 (log 2fc=−2.3) transcripts are also significantly overexpressed, implying induction of the EMT in the responder tumor.

As shown for pancreatic cancer, suppression of EMT concomitant with galunisertib administration could increase the sensitivity of the the responder tumor to the drug and thus would be an interesting option for a combination therapy.^19^

## Discussion

Personalized therapy is one of the biggest challenges to overcome successfully the heterogeneity of HCC and to offer patients the most effective treatment for those recipients who could not receive LT, LRTs or hepatic resection.^20^ This study firstly demonstrates that tailoring treatment strategies according to the individual HCCs genetic profile provides a wealth of therapeutic choices going far beyond standard sorafenib treatment for liver cancers. Here, we demonstrate that neither of the HCC tissues, responder and non-responder to galunisertib effectiveness *in vitro* according to our previous investigation,^15^ contained the somatic mutations most frequently found in HCCs, such as TERT or TP53 mutations.^21^

However, the mutation that we suspect has the largest impact on the differential character of molecular patterns in the two tumors is the mutation in the *TGFBR3* gene in the non-responder tumor. In HCCs as in other cancers such as ovarian cancer, TGFBR3 may function as a cancer suppressor,^12^ and in pancreatic cancer loss of TGFBR3 expression promoted cancer progression.^22^ In a pancreatic cancer model of the EMT, TGFBR3 suppresses the associated increased motility and invasiveness. Suppression of motility and invasiveness does not depend on its cytoplasmic domain or its coreceptor function but is mediated by ectodomain shedding, generating soluble TGFBR3.^23^ Since it has been shown that AXL activates autocrine TGFB signaling in HCCs, resulting in a poor prognosis, it is tempting to speculate that in the non-responder tumor – due to the mutation in the *TGFBR3* gene – the TGFB autocrine loop including AXL is blocked.^24^ This is consistent with our previous observation whereby TGF-*β* circulating levels did not correlate with the staging of the disease.^25^ The observed mutation of the *TGFBR3* gene in the non-responder tumor may be indicative of a certain type of HCC and at the same time may serve as a biomarker for companion diagnostics of TGFBR1-targeting drugs like galunisertib.^26^ Moreover, these results indicate that TGFBR3 may be a similarly promising drug target (not necessarily inhibitor) as compared with the TGFB receptors, because its mutation seemingly has pleiotropic effects on TGFB-related pathways. The imbalance of the TGF pathway may evoke several downstream effects, including the proteolytic remodeling of the ECM proteins by MMPs and the EMT, both leading to the progression of the cancer.^27^ The modulation of the immune response has been reported also to be under the control of TGFB, although here we cannot decide whether the marked differences between immune-related reactome pathways that also distinguish the responder from the non-responder tumor are another consequence of the difference in TGFB signaling or not. In any case, the differential control of the immune system by the two tumors would have a major impact on a potential combination therapy if it should include immune-modulating drugs. These showed promising results and even a complete response in cancers with an otherwise poor prognosis.^27^ Thus, the responder would probably profit most from blocking PD-1 checkpoint inhibition by drugs such as nivolumab, ipilimumab or pembrolizumab, if necessary supported by STINGVAX treatment. In lung cancers, anti-PD-1 therapy profited from increased nonsynonymous mutations, especially in DNA repair pathways in comparison with tumors with lower mutation frequencies. It was reported that 69% of lung cancer patients with a high mutation frequency experienced durable clinical benefits from PD-1 blockade as compared with 20% of patients with fewer mutations.^28^ High mutation rates were also significantly associated with progression-free survival. Similar results were reported by Rizvi *et al.*,^29^ who noted that this favorable result was at least in one case related to neoantigen-specific CD8+ T-cell responses, suggesting that anti-PD-1 therapy enhances neoantigen-specific T-cell reactivity. The number of protein changing mutations for our HCC tumors was 275 in the responder and 226 in the non-responder tumor. Thus, both our tumors contain considerably more somatic mutations than the lung cancers studied by the above authors. This larger number of mutations opens up the possibility to load synthetic RNAs coding for a large number of different tumor-derived neoantigens via intravenously administered RNA–lipoplexes onto the immune system where they may evoke effector and memory T-cell responses, and mediate IFN*α*-dependent rejection of tumors.^30^ Alternatively, synthetic naked DNA can be administered to skin-resident dendritic cells via micropinocytosis, which then prime antigen-specific CD8+ T cells.^31^

Whereas the responder tumor engages PD-1 checkpoint inhibition, the non-responder tumor has already upregulated the 'CLEC7A/inflammasome pathway'. Thus, the non-responder would probably be best treated using a combination therapy that boosts its already existing readiness to fight foreign invaders by such approaches as vaccination with the Bacille Calmette-Guerin tuberculosis, which in the case of this patient may not only help protect against pathogens but also against the tumor.^32^ The upregulation of the CLEC7A pathway may be exploited for therapeutic, anticancer overactivation of the IL-6/STAT3 influenced innate immune system by components of pathogen cell walls such as fungal *β*-1,3 glucans or other immune-stimulatory substances. These would not only (over)activate the CLEC7A pathway but also specific, TLR cascades upregulated in the non-responder tumor.^33, 34, 35^ This immune-stimulatory strategy could also benefit from the knowledge of the mutated proteins in the tumor. These could be used to synthesize candidate-mutated T-cell epitopes that may be identified using a major histocompatibility complex-binding algorithm for recognition by tumor-infiltrating lymphocytes. CLEC7A drives IL-1B biogenesis and maturation through a noncanonical caspase-8-dependent inflammasome in the host innate immune system.^36^ The receptor dimerizes upon ligand binding and phosphorylation by kinases of the SRC family. Activation of CLEC7A finally leads to the activation of transcription factor NF-*κ*B, which then induces the production of inflammatory cytokines and chemokines such as TNF, IL-23, IL-6 or IL-2.^37^ Dectin-1 reduced hepatic fibrosis and hepatocarcinogenesis by negative regulation of TLR4 signaling pathways.^38^ In conclusion, the study we present here has the aim of contributing to the discussion as to how therapeutic options arising from our growing ability to characterize individual cancers at the molecular level may best be used to the benefit of the individual patient.

## Materials and methods

### *Ex vivo* HCC tissue profiling and DNA–RNA extraction

Tumoral and peritumoral tissues were isolated collecting the peritumoral at least at 4–5 cm by the limit of the lesion. *Ex vivo* assay was performed as described previously.^15^ Briefly, human HCC samples were cultured for 48 h in serum-free condition in the presence of galunisertib and TGF-*β*. Tissues were stored in liquid nitrogen before RNA–DNA isolation. Total RNA and DNA were isolated using the AllPrep DNA/RNA Mini Kit (Qiagen, Hilden, Germany) according to the supplier’s protocol.

### Massive analysis of cDNA ends

MACE libraries were prepared from the two peritumoral and the two tumor tissues using the MACE Kit (GenXPro GmbH, Frankfurt Germany) according to the supplier’s protocol as described.^16^ In short, polyadenylated mRNA was extracted (Dynabeads mRNA Purification Kit; Life Technologies, Whaltham, MA, USA) from 5 *μ*g total RNA (large RNA fraction) and reverse transcribed with biotinylated oligo (dT) primers. cDNA was prepared and fragmented to an average size of 250 bp by sonification using a Bioruptor (Diagenode, Seraing, Belgium). Biotinylated cDNA 3′ ends were captured by streptavidin beads and bound to TrueQuant DNA adapters (Waltham, MA, USA) provided in the kit. The libraries were amplified by PCR, purified by SPRI beads (Agencourt AMPure XP; Beckman Coulter, Brea, CA, USA) and sequenced (NextSeq 500; Illumina Inc., San Diego, CA, USA).

### Bioinformatics analysis of MACE data

A total of ~114.12 million MACE sequencing reads was obtained from the four cDNA sequencing libraries. All PCR-duplicated reads identified by the TrueQuant technology were excluded from the raw data sets. The remaining reads were further quality trimmed and the poly (A)-tail was clipped off. Filtered reads were aligned to the human reference genome (hg38) using the *bowtie2* mapping tool.^39^ Unmapped reads were then aligned to the transcriptome assembly using the same mapping tool. Finally, the respective bam files of each MACE library were merged into respective single combined bam files using SAMtools.^40^ The hg38 refSeq annotation GTF file, which includes coding genes as well as long noncoding RNAs (http://genome.ucsc.edu/cgi-bin/hgTables), was imported into the htseq-count annotation tool to annotate merged bam files. This yielded a set of combined enriched gene count data as output. Next, normalization of enriched gene count data followed by differential gene expression analysis between matched tumor samples and peritumor samples were performed to estimate expression levels of 41 636 genes/transcripts and 432 long noncoding RNAs, using the DeSeq R/Bioconductor package. To account for multiple testing, the false discovery rate (FDR) was estimated according to Klipper-Aurbach *et al.*^41^ Genes with *P*-values < 0.01, and an absolute log 2 fold change |(log 2fc)| > 2 were considered differentially expressed. Differentially expressed genes were uploaded to the Database for Annotation, Visualization and Integrated Discovery (DAVID) using an enrichment cutoff of FDR < 0.05.^42^ Differentially expressed genes were further assigned to biological pathways using the Reactome database, a curated knowledgebase of biological pathways in humans.^43^ Immune-related pathways were separated out from the entire list of reactome pathway terms based on evidence collected from recently published literature and terms were linked with immune regulation. Then, immune pathways in both responder and non-responder samples were ranked via estimating the percentage of affected genes in a particular pathway. The STRING database v.10^44^ was used with standard parameters to discover interactions between proteins encoded by genes that were significantly up- or downregulated or somatically mutated in the tumor, respectively.

### Identification of drugs targeting kinases in reactome pathways enriched in upregulated genes

Kinases engaged in pathways enriched with upregulated genes in tumor tissues were identified through mapping via their reactome ID, followed by extraction of kinase genes from mapped reactome IDs. Those kinases that were upregulated at log 2fc>+1 and with a *P*-value<0.05 in both responder and non-responder tumor samples were chosen for further drug enrichment/interaction. Drug interaction analysis was performed by interrogating the Drug-Gene Interaction Database (DGIdb), a database and web interface for ascertaining known and potential drug–gene relationships applying standard parameters.^45^

### Exome library preparation, sequencing and analysis

DNA was quantified using Qubit HS dsDNA assay (Life Technologies). Exomes were captured from a total of 50 ng of DNA using Illumina's Nextera Custom Target Enrichment Kit (Illumina) according to the Illumina Nextera Rapid Capture Enrichment Guide (August 2013) . A total of 2 × ~121.2 million 151 bp long paired-end (PE) reads of tumoral and peritumoral tissues was generated using a NextSeq platform (Illumina). Initially, raw reads were checked for adaptors and low-quality bases. The remaining ~2 × 117.64 million PE reads were aligned to the human genome (hg38) reference sequence using the *bwamem* mapping tool with default settings. The Picard Toolkit (San Francisco, CA, USA) was used to preprocess mapped bam files in which *SortSam*, *MarkDuplicate* and *BuildBamIndex* commands were primarily used before calling for variants including SNPs and InDels. The standard GATK pipeline (http://www.broadinstitute.org/gatk/) was used to call variants, in which the first step was the local realignment of reads around InDels and SNPs using *IndelRealigner* and *RealignerTargetCreator* commands, respectively, to curtail the number of mismatching bases across all the reads. Thereafter, a *Mutect2* command^46^ was used to identify somatic SNPs and the *HaplotypeCaller* command was used to detect somatic InDels. We removed low-quality SNPs by applying high-stringency criteria using the *VariantFiltration* command with filtering options (QD<2.0||FS>60.0||MQ<40.0||HaplotypeScore>13.0||MQRankSum<−12.5||ReadPosRankSum<−8.0, where || indicates OR). In the same way, low-quality InDeLs were removed with filtering options (QD<2.0||FS>200.0||ReadPosRankSum<−20). Remaining high-quality somatic SNPs and InDels were annotated using *SnpEff*^47^ to assign them to different gene-related categories such as nonsynonymous, exonic and so on. The STRING database v.10^44^ was used again with standard parameters to discover interactions between gene products (proteins) encoded by genes that harbor protein-changing SNPs/InDels.

### Identification of potential therapeutic targets and corresponding drug candidates using the MPD drug prediction tool

Drug enrichment analysis was performed by calculating the significance of overlaps between a subset of 625 FDA-approved druggable genes (obtained from http://www.proteinatlas.org), which are significantly upregulated in one or the other tumor tissue (thresholds: *P*-value<0.05, log 2fc>1.0). Moreover, they are enriched in overall drug interactions for each compound as compared with all genes in the human genome. *P*-values denoting the significance of the enrichment were calculated using hypergeometric distribution. Drug interactions were annotated using data from DGIdb (http://dgidb.genome.wustl.edu).

## Figures and Tables

**Table 1:** Drugs targeting at least six best druggable upregulated genes suggested for treatment of the responder tumor by the MPD pipeline

**ID**	*P***-value**	**FDR**	**Observed overlap**	**Description**	**Indication**
Regorafenib	0	0	PDGFRB, DDR2, EPHA2, KIT, BRAF, MAPK11, FLT4, FGFR1, KDR, FLT1 Count: 10	Regorafenib is an orally administered inhibitor of multiple kinases. It is used for the treatment of metastatic colorectal cancer and advanced gastrointestinal stromal tumors. FDA approved on 27 September 2012	Regorafenib is indicated for the treatment of patients with metastatic CRC who have been previously treated with fluoropyrimidine, oxaliplatin and irinotecan, an anti-VEGF therapy, and, if KRAS wild type, an anti-EGFR therapy. Regorafenib is also indicated for the treatment of patients with locally advanced, unresectable or metastatic GIST who have been previously treated with imatinib mesylate and sunitinib malate.
Dasatinib	0	0	PDGFRB, SRC, DDR2, EPHA2, KIT, BRAF, ABL1, LCK, YES1 Count: 9	Dasatinib is an oral dual BCR/ABL and Src family tyrosine kinase inhibitor approved for use in patients with CML. The main targets of dasatinib are BCR/ABL, SRC, ephrins and GFR.	For the treatment of adults with chronic, accelerated, or myeloid or lymphoid blast phase chronic myeloid leukemia with resistance or intolerance to prior therapy. Also indicated for the treatment of adults with Philadelphia chromosome-positive acute lymphoblastic leukemia with resistance or intolerance to prior therapy.
Sorafenib	0	4E−10	PDGFRB, KIT, MAPK3, MAPK1, MAPK11, FLT4, BRAF, KDR, FLT1 Count: 9	Sorafenib (rINN), marketed as Nexavar by Bayer, is a drug approved for the treatment of advanced renal cell carcinoma (primary kidney cancer). It has also received 'Fast Track' designation by the FDA for the treatment of advanced hepatocellular carcinoma, and has since performed well in phase III trials. Sorafenib is a small molecule inhibitor of Raf kinase, PDGF, VEGF receptor 2 and 3 kinases and c-Kit, the receptor for stem cell factor. The originality of Sorafenib lays in its simultaneous targeting of the Raf/Mek/Erk pathway.	Sorafenib is indicated for the treatment of unresectable hepatocellular carcinoma and advanced renal cell carcinoma.
Iloprost	0	2E−10	PTGIR, PTGER2, PLAT, PDE4A, PDE4B, PDE4C, PDE4D Count: 7	Iloprost is a synthetic analog of prostacyclin PGI2. Iloprost dilates systemic and pulmonary arterial vascular beds. It is used to treat PAH	Used for the treatment of pulmonary arterial hypertension.
Ibudilast	0	4E−10	PDE3A, PDE4A, PDE4B, PDE4C, PDE4D, IL-1B Count: 6	Ibudilast is an anti-inflammatory and neuroprotective oral agent that shows an excellent safety profile at 60 mg per day and provides significantly prolonged time-to-first relapse and attenuated brain volume shrinkage in patients with RR and/or SP MS. Ibudilast is currently in development in the United States (codes: AV-411 or MN-166), but is approved for use as an anti-inflammatory in Japan.	For the treatment of multiple sclerosis, asthma and cerebrovascular disease.

Abbreviations: CML, chronic myelogenous leukemia; CRC, colorectal cancer; EGFR, epidermal growth factor receptor; FDR, false discovery rate; GIST, gastrointestinal stromal tumor; MS, multiple sclerosis; PAH, pulmonary arterial hypertension; PDGF, platelet-derived growth factor; RR, relapsing-remitting, SP, secondary progressive; VEGF, vascular endothelial growth factor

**Table 2:** List of TKIs targeting the upregulated kinases in the responder tissue

**Responder kinase**	**Substance**	**Class**	**Target application**	**Status**
*PDK1*	Dichloroacetic acid	Inhibitor	Arthritis, ankylosing spondylitis and menstrual pain	Experimental
*BTK*	Ibrutinib	Inhibitor	Mantle cell lymphoma, chronic lymphocytic leukemia	Approved
*AKT3*	Ipatasertib (GDC-0068)	Inhibitor	Front-line for metastatic triple-negative breast cancer	Phase II
	MK-2206	Inhibitor	Endometrial serous cancer	Phase II
	Omipalisib (GSK2126458)	Inhibitor	Solid tumors, idiopathic pulmonary fibrosis	Phase I
	AZD5363	Inhibitor	Solid tumors with AKT/PIK3CA mutations	Phase I
*MAPK3*	Purvalanol	Inhibitor		Experimental
*SYK*	Ellagic acid	Inhibitor	Solid tumors	Natural compound
	HMPL-523	Inhibitor	Targeted B-cell receptor signaling therapy for autoimmune diseases including rheumatoid arthritis, systemic lupus erythematosus and allergy, as well as hematological cancers (i.e. B-cell malignancies) including lymphoma and leukemia	Phase I
*PRKCA*	Quercetin	Inhibitor	Solid tumors	Natural compound
	Ellagic acid	Inhibitor/competitive	Solid tumors	Natural compound
	Midostaurin	Inhibitor	Patients older than 18 years with FLT3-mutated AML	Approved
	Sotrastaurin acetate	Inhibitor	Metastatic uveal melanoma	Phase I
*PRKCD*	Quercetin	Inhibitor	Solid tumors	Natural compound
	Delcasertib (KAI-9803)	Inhibitor	Stroke	Experimental
*PIK3R2*	Quercetin	Inhibitor	Solid tumors	Natural compound
	Apitolisib (GDC-0980)	Inhibitor	Renal carcinoma	Phase II
	Gedatolisib (PKI-587)	Inhibitor	Recurrent endometrial cancer	Phase II
	GSK2636771	Inhibitor	Advanced refractory solid tumors, lymphomas, metastatic castration-resistant prostate cancer	Phase I/II
	Duvelisib	Inhibitor	Inhibitor of PI3K delta and gamma for hematologic malignancies and inflammatory conditions	Phase III
	SF1126	Inhibitor	Orphan drug for B-cell chronic lymphocytic leukemia	Phase II
	XL147	Inhibitor	Endometrial carcinoma	Phase II
	TGR 1202	Inhibitor	In combination with ublituximab for chronic lymphocytic leukemia, non-Hodgkin's lymphoma	Phase I/II
*JAK3*	Tofacitinib	Inhibitor	Rheumatoid arthritis	Approved in Switzerland

Abbreviations: AML, acute myeloid leukemia; FLT3, FMS-like tyrosine kinase 3; PI3K, phosphoinositide 3-kinase

**Table 3:** Drugs targeting at least four best druggable upregulated genes suggested for treatment of the non-responder tumor by the MPD pipeline

**ID**	***P*-value**	**FDR**	**Observed overlap**	**Description**	**Indication**
Marimastat	2E−10	0	MMP14, MMP17, MMP10, MMP12, MMP9, MMP1 Count: 6	Used in the treatment of cancer, marmiastat is an angiogenesis and metastasis inhibitor. As an angiogenesis inhibitor it limits the growth and production of blood vessels. As an antimetatstatic agent it prevents malignant cells from breaching the basement membranes.	For the treatment of various cancers
Celecoxib	1.38E−08	2.1E−05	PTGS2, CACNB3, CACNA1C, KCNQ3, VEGFA, MMP9 Count: 6	Celecoxib is a NSAID used in the treatment of osteoarthritis, rheumatoid arthritis, acute pain, painful menstruation and menstrual symptoms, and to reduce numbers of colon and rectum polyps in patients with familial adenomatous polyposis. It is marketed by Pfizer under the brand name Celebrex. In some countries, it is branded Celebra. Celecoxib is available by prescription in capsule form.	For relief and management of OA, RA, JRA, ankylosing spondylitis, acute pain, primary dysmenorrhea and oral adjunct to usual care for patients with familial adenomatous polyposis.
Ibudilast	2.4E−09	7.3E−06	PDE4A, IL-1B, PDE4C, PDE4B Count: 4	Ibudilast is an anti-inflammatory and neuroprotective oral agent that shows an excellent safety profile at 60 mg per day and provides significantly prolonged time-to-first relapse and attenuated brain volume shrinkage in patients with RR and/or SP MS. Ibudilast is currently in development in the United States (codes: AV-411 or MN-166), but is approved for use as an antiinflammatory in Japan.	For the treatment of multiple sclerosis, asthma, and cerebrovascular disease.
Dyphylline	1.12E−08	2.3E−05	PDE7A, PDE4A, PDE4B, PDE4C Count: 4	A theophylline derivative with broncho- and vasodilator properties. It is used in the treatment of asthma, cardiac dyspnea and bronchitis (PubChem).	For relief of acute bronchial asthma and for reversible bronchospasm associated with chronic bronchitis and emphysema.
Urokinase	3.35E−08	3.4E−05	PLAUR, SERPINB2, SERPINE1, PLAU Count: 4	Low-molecular-weight form of human urokinase, which consists of an A chain of 2000 Da linked by a sulfhydryl bond to a B chain of 30 400 Da. Recombinant urokinase plasminogen activator.	Urokinase can be used for the treatment of pulmonary embolism, coronary artery thrombosis, i.v. catheter clearance, and venous and arterial blood clots.
Ketotifen	3.35E−08	4E−05	PDE7A, PDE4A, PDE4B, PDE4C Count: 4	A cycloheptathiophene blocker of histamine H1 receptors and release of inflammatory mediators. It has been proposed for the treatment of asthma, rhinitis, skin allergies and anaphylaxis (PubChem).	Indicated as an add-on or prophylactic oral medication in the chronic treatment of mild atopic asthmatic children. Also used as self-medication for the temporary relief of itching of the eye due to allergic conjunctivitis (ophthalmic).

Abbreviations: FDR, false discovery rate; i.v., intravenous; JRA, juvenile rheumatoid arthritis; MS, multiple sclerosis; NSAID, non-steroidal anti-inflammatory drug; OA, osteoarthritis; RA, rheumatoid arthritis; RR, relapsing-remitting; SP, secondary progressive

**Table 4:** Upregulated kinases engaged in a number of pathways enriched in upregulated genes in the non-responder tumor tissue

**Kinase in non-responder**	**No. of enriched pathways**	**Drug**	**Interaction type**	**Application**
MAP3K7	4	RGB-286638	Inhibitor	Solid tumors/phase I
IRAK2	3	−/−	−/−	
MAP2K6	3	Trametinib	Inhibitor	Unresectable or metastatic melanoma with a BRAF V600E or V600K mutation
		Selumetinib	Inhibitor	Adjuvant treatment of patients with stage III or IV DTC
		Dabrafenib mesylate	Inhibitor	Single agent treatment for patients with BRAF V600E mutation-positive advanced melanoma
RIPK2	3	SRC kinase inhibitor I	N/A	
MAPK11	2	Regorafenib	Inhibitor	mCRC
RIOK1	2	−/−	−/−	
*CDK4*	1	−/−	−/−	
MAP3K8	1	MEK inhibitor II	N/A	
		TPL2 kinase inhibitor	N/A	

Abbreviations: DTC, differentiated thyroid cancer; mCRC, metastatic colorectal cancer; N/A, not applicable; TKI, tyrosine kinase inhibitor

TKIs targeting the respective kinases are also indicated

**Table 5:** Top enriched immune-related reactome pathways comprising genes upregulated in one or the other tumor tissue

**Immune-related pathway**	**Genes in pathway**	**Responder**	**Non-responder**
		**% upregulated genes in pathway**	**% upregulated genes in pathway**
PD-1 signaling	45	20	0
Regulation of innate immune response to cytosolic DNA	22	18.18	4.55
TRIF-mediated TLR3/TLR4 signaling	108	17.59	0
Diseases of immune system	35	17.14	0
Cytokine signaling in immune system	747	16.73	3.48
DAP12 interactions	401	15.46	3.49
CTLA4 inhibitory signaling	26	15.38	0
DAP12 signaling	384	15.36	3.39
Immune system	1984	14.26	3.38
TLR6:TLR2 cascade	107	14.02	7.48
TLR2 cascade	110	13.64	7.27
TLR1:TLR2 cascade	110	13.64	7.27
Adaptive immune system	1075	13.21	3.07
Innate immune system	1064	12.97	3.29
STING-mediated induction of host immune responses	25	12	4
Regulation of complement cascade	30	6.67	10
Complement cascade	211	1.42	1.42
Classical antibody-mediated complement activation	165	0.61	0
CLEC7A/inflammasome pathway	8	0	37.5
TLR10 cascade	91	0	8.79
TLR5 cascade	91	0	8.79
TLR3 cascade	108	0	8.33
Activated TLR4 signaling	128	0	7.03
TLR4 cascade	141	0	6.38
TLR cascades	163	0	6.13
MHC class II antigen presentation	141	0	4.26
CLRs	182	0	3.3
TCR signaling	145	0	2.76
Signaling by the BCR	383	0	2.61
Class I MHC-mediated antigen processing and presentation	408	0	2.45

Abbreviations: BCR, B-cell receptor; CLR, C-type lectin receptor; CTLA4, cytotoxic T-lymphocyte-associated protein 4; MHC, major histocompatibility complex; STING, stimulator of interferon gene; TLR2, Toll-like receptor 2; TLR3, Toll like receptor 3; TLR4, Toll-like receptor 4; TLR5, Toll-like receptor 5; Toll-like receptor 10; TRF, TRIF-domain-containing adapter-inducing interferon-*β*

**Table 6:** Number of mutations identified by whole-exome sequencing in both the responder and non-responder tumor and their location in relation to genes

**Location of mutation**	**Responder**	**Non-responder**
Downstream	36	12
Exon	29	11
Intergenic	145	143
Intron	199	203
NA	2	4
Protein changing_coding	275	226
Synonymous_coding	88	56
Upstream	51	77
UTR_3_prime	22	8
UTR_5_prime	2	0
Indels	38	53
